# *Chlamydia trachomatis* seropositivity among women with tubal factor infertility and fertile controls: a comparative study

**DOI:** 10.11604/pamj.2023.44.178.29443

**Published:** 2023-04-14

**Authors:** Oluwaseyi Isaiah Odelola, Adebayo Adekunle Akadri

**Affiliations:** 1 Department of Obstetrics and Gynaecology, Olabisi Onabanjo University Teaching Hospital Sagamu, Ogun State, Nigeria,; 2 Department of Obstetrics and Gynaecology, Babcock University, Ilishan-Remo, Ogun State, Nigeria

**Keywords:** *Chlamydia trachomatis*, antibody titre, tubal factor infertility, hysterosalpingography, laparoscopy

## Abstract

**Introduction:**

Chlamydia trachomatis is the most reported bacterial sexually transmitted infection and if not properly treated may lead to tubal blockage. Tubal factor infertility is the most common form of infertility in Nigeria. This study was designed to determine the usefulness of chlamydia antibody testing in diagnosis of tubal factor infertility.

**Methods:**

this was a comparative cross-sectional study conducted in Olabisi Onabanjo University Teaching Hospital Sagamu. One hundred and forty-seven women with tubal blockage on hysterosalpingography and confirmed with laparoscopy, and pregnant control were recruited using convenience sampling method. Information obtained and chlamydia assay results were entered into a computer and analyzed using SPSS version 21. Chi-square was used to determine association between categorical variables. Logistic regression analysis was used to determine the risk factors associated with chlamydia infection.

**Results:**

ninety-four (63.9%) of the women with tubal factor infertility were positive for chlamydia IgG antibodies while 37(25.2%) women in the control group had positive results for IgG antibody. This was statistically significant (P=0.001). Analysis using multivariate logistic regression shows early age of coitarche, presence of multiple sexual partners and previous sexually transmitted infection were significantly associated with chlamydia infection (P=0.001).

**Conclusion:**

there was a strong association between chlamydia seropositivity and tubal blockage. Early age at coitarche, previous sexually transmitted infection and multiple sexual partners are significant risk factors for chlamydial infection. Chlamydia trachomatis antibody testing could be used as marker for tubal blockage when evaluating infertile patient.

## Introduction

Infertility is a major life event associated with profound implications on the reproductive health of women [1]. It is associated with a wide range of socio-cultural, emotional, physical and financial problems. Infertility is the failure to achieve a clinical pregnancy after 12 months of regular unprotected sexual intercourse [1,2]. About 70 million couples worldwide experience infertility [3,4]. The prevalence of infertility is highest in developing countries including sub-Saharan Africa, with rates ranging from 20-46% [5,6]. In Nigeria, infertility accounts for about 60% of gynaecological consultations [3]. The aetiology of infertility in females is often classified under tuboperitoneal factor, ovulatory factor, uterine factor, and cervical factor [7,8]. Tuboperitoneal factor is implicated in 43% of cases of infertility in Nigeria and most (58.5%) fallopian tube damage is due to chronic pelvic inflammatory disease [9]. *Neisseria gonorrhea* and *Chlamydia trachomatis* are mostly implicated in Pelvic inflammatory disease [10]. Other organisms implicated are *Mycoplasma genitalium, Mycoplasma hominis, Ureaplasma spp, viellomella spp*, and other genital tract endogenous anaerobes and facultative bacteria [10-12]. *Chlamydia trachomatis* is associated with at least 50% of acute pelvic inflammatory disease and is asymptomatic in 70-80% of cases [13]. The incidence of *Chlamydia trachomatis* is increasing worldwide [14,15]. It causes intraluminal adhesions, fibrosis and tubal blockage which will ultimately have adverse effect on the fertility of affected woman.

Many studies have reported association between chlamydia antibody titre and the risk of tubal infertility [16,17]. Common investigative procedures for assessing tubal patency include hysterosalpingography (HSG), laparoscopy with chromopertubation and hystero-contrast-sonography. However, these tests are invasive, painful and may require analgesia and anaesthesia. Laparoscopy is considered the gold standard in tubal assessment, particularly in detecting peritubal adhesions and endometriosis; [19] however, it is an invasive and relatively expensive procedure. HSG is cheaper than laparoscopy and is often considered as a substitute, however, it is also invasive and has suboptimal sensitivity (65%) and specificity (83%) [16,18]. chlamydia antibody testing is cheap, easy to perform and less invasive with minimal inconvenience [19]. *Chlamydia trachomatis* antibody testing can be part of work up for infertile couples or serve as basis for triaging to determine those that will require laparoscopy for further evaluation. This study was designed to determine the association between chlamydia antibody positivity and tubal infertility.

## Methods

This was a comparative cross-sectional study conducted in Olabisi Onabanjo University Teaching Hospital (OOUTH), a tertiary institution located in the semi-urban town of Sagamu, Ogun State South Western geopolitical zone of Nigeria. The hospital serves as a referral centre for Obstetric and Gynaecological services from neighbouring towns and villages of Ogun State and Lagos State. The study was carried out between 1^st^ March, 2018 and 30^th^ July, 2020. The sample size was calculated based on the known prevalence rate of 38.6% and 22.8% of chlamydia immunoglobulin G antibody among infertile and fertile women respectively in Calabar South-South Nigeria [19]. The sample size was determined using the formulae for comparing proportions in a cross-sectional study [20].

n= (r+1)/r (p*)(1-p*)(Z_ß_-Z_a/2_)2 / P1-P2

Assuming the standard normal variate for 80% power is 0.84 and standard normal variate for 0.05% level of significance 1.96, the minimum sample size for each group was 134. However, to allow for possible incomplete data, 10% was added giving a final sample size of 147 in each arm was used for the study. The study participants were infertile women with bilateral tubal blockage on hysterosalpingography and confirmed with laparoscopy. Women with evidence of endometriosis on laparoscopy, those with previous pelvic surgery including salpingectomy and women who got pregnant either spontaneously after treatment for infertility or through assisted reproductive technology were all excluded from the study. The participants were recruited consecutively until the sample size was reached. Pregnant women were also recruited consecutively at the antenatal clinic to serve as the control group. The study participants were counseled and written consent obtained.

**Specimen collection:** blood samples were collected under aseptic conditions from a vein on the dorsal surface of the non-dominant hand or in the antecubital fossa of the non-dominant upper extremity after cleaning with 70% ethyl alcohol. About 5mls of blood was drawn into a clean sterile plain plastic bottle. The blood was centrifuged for over five minutes and the serum sample obtained was stored in the refrigerator at temperatures of 4°C to 8°C until analysis was done. The samples were analysed in the Department of Microbiology of Olabisi Onabanjo University Teaching Hospital.

**Procedure for the assay**

The serological assays were done using enzyme linked immunosorbent assay (ELISA) kit manufactured by DIA PRO LTD (Milano, Italy). The ELISA *Chlamydia trachomatis* IgG kit is used for both quantitative and qualitative determination of IgG antibody specific for *Chlamydia trachomatis* in human plasma and sera. The test is an indirect solid phase enzyme linked immunoassay with 96 well microplates that had been pre-coated with an immunodominant specific polypeptide derived from *Chlamydia trachomatis* major outer-membrane antigen. This made the assay very specific for *Chlamydia trachomatis* thereby avoiding cross-reaction with other chlamydia serovars. The reagent test kit was brought to room temperature after removal from the refrigerator at a temperature of 4°C-8°C. At the time of analysis, samples were allowed to thaw and were made into 1: 101 dilution (10µl of sera with 1000µl of diluents) on a microplate consisting of 96 microwells incubated at 37°C. At this stage, anti-C trachomatis IgG was captured when present on the solid phase. The microplates were washed off other components of the sample with a microplate washer, following which 100µl of enzyme conjugate (anti IgG antibody labelled with peroxidase) was put in each well, sealed and incubated for 60 minutes at 37°C. The microwell was again washed with automatic washer. One hundred microlitres (100µl) of the substrate was pipetted into each well. The antibody-enzyme conjugate captured on the solid phase acted on the substrate to generate an optical signal that is proportional to the amount of anti C trachomatis antibody present. After 20 minutes of incubation at room temperature, 100µl of sulphuric acid were put into all the wells and the colour intensity of the solution of each well was measured photometrically at 450nm by ELISA reader. Samples with concentration higher than 5arbU/ml (5 arbitrary units per milliliter) were considered positive for IgG antibody.

**Data collection:** data was collected with the aid of a questionnaire developed specifically for this study. The questionnaire was pretested among patients with infertility who had presented to State hospital Ijebu-Ode, Nigeria (a nearby State-owned secondary health facility). Findings from the pre-test allowed for modifications of some aspects of the study instrument. The questionnaire comprised of three sections. The first section was used to obtain information on the sociodemographic characteristics of study participants (age, ethnicity marital status, religion, educational status, and occupation). Information on the last menstrual period, parity, and history of risk factors for tubal infertility (such as age at coitarche, number of sexual partners, history of previous treatment for sexually transmitted diseases) were recorded in the second section of the questionnaire. History of symptoms of chlamydia infections, results of serum chlamydia antibody testing and results of tubal patency tests (hysterosalpingography and laparoscopy) were also recorded in the third section of the questionnaire. The outcome variable of the study was the serum chlamydia antibody determined using ELISA technique.

### Data management and analysis

All the information obtained from the questionnaire and the results of chlamydia antibody assay were entered into a computer and analysed using the Statistical Package for Social Science for Windows software version 21(Armonk, NY: IBM, 2015). Categorical variables such as sociodemographic characteristics, type of infertility and chlamydia screening results were summarized using frequencies and percentages. Continuous variable such as age was summarized with mean and standard deviation. Chi-square test and Relative Risk were calculated to determine association between and presence of tubal blockage, type of infertility (primary or secondary); and chlamydia seropositivity. Variables such as age of coitarche, parity, previous Sexually Transmitted Infections (STIs) and number of life time sexual partners were included in the logistic regression model to determine probable risk factors for chlamydia infection. Differences were considered statistically significant with p value less than 0.05. Ethical approval for the study was obtained from health research and ethics committee of Olabisi Onabanjo University Teaching Hospital Sagamu (reference number: OOUTH/HREC/173/2017).

## Results

One hundred and forty-seven women with tubal infertility were included as cases while another 147 pregnant women served as control. The mean age (±SD) of the women with tubal infertility was 29.44 ± 5.10 years while that of the control was 29.62 ± 4.93 years. The two groups were comparable in terms of age. In both cases and control groups, majority of the respondents were within the age group of 21-30 years followed by age group 31-40 years. Majority of the participants were of Yoruba ethnicity accounting for 112(85.0%) and 129(87.8%), respectively, for cases and controls. The educational status of the infertile women and control group were comparable (X^2^=0.539, P=0.910). As regards the marital status of the cases, 142(96.6%) were married whereas all the pregnant controls were married. Most of the respondents were artisans, 37(25.2%) and 34(23.1%) for cases and controls respectively (Table 1). Table 2 shows that 94 (63.9%) women with tubal factor infertility were positive for chlamydia IgG antibodies while 37(25.2%) women in the control group had positive results for IgG antibody. This pattern indicates that women with tubal infertility had statistically significant higher prevalence of chlamydia seropositivity compared to the control group [RR 2.2, 95% CI: 1.7 -2.8; P< 0.001].

**Table 1:** sociodemographic characteristics of the study population

Sociodemographics	Case (%) n = 147	Control (%) n = 147	Chi square value	p value
**Age**				
21-30	94(63.9)	87(59.2)	1.406	0.495
31-40	46(31.3)	55(37.4)		
>40	7(4.8)	5(3.4)		
**Ethnicity**				
Yoruba	112(76.2)	123(83.7)	5.282	0.152
Igbo	16(10.9)	11(7.5)		
Hausa/Fulani	6(4.1)	8(5.4)		
Others	13(8.8)	5(3.4)		
**Religion**				
Christian	85(57.8)	92(62.6)	1.002	0.606
Islam	59(40.2)	51(34.7)		
Traditional	3(2.0)	4(2.7)		
**Educational Status**				
Informal Primary	11(7.5) 31(21.1)	13(8.8) 29(19.7)	0.539	0.910
Secondary	58(39.5)	54(36.7)		
Tertiary	47(32.0)	51(34.7)		
**Marital Status**				
Single	2(1.4)	0(0.0)	5.086	0.079
Married	142(96.6)	147(100.0)		
Divorced	3(2.0)	0(0.0)		
**Occupation**				
Civil Servant	29(19.7)	31(21.1)	0.514	0.972
Professional	27(18.4)	30(20.4)		
Artisan	37(25.2)	34(23.1)		
Trader	34(23.1)	31(21.1)		
Unemployed	20(13.6)	21(14.3)		
**Parity**				
0	87(59.2)	35(23.8)	58.711	0.001
1	46(31.3)	43(29.3)		
≥2	14(9.5)	69(46.9)		

**Table 2:** the association of chlamydial seropositivity and tubal infertility

Variables	Case (%) n=147	Control (%) n=147	Relative Risk (95% CI)	p value
**Chlamydia Screening**				
Positive	94(63.9)	37(25.2)	2.2(1.7 -2.8)	<0.001
Negative	53(36.1)	110(74.8)		

Table 3 depicts that out of the 147 cases of tubal factor infertility, twenty-six (17.7%) had primary infertility while 121(82.3%) had secondary infertility. The proportion of chlamydia seropositivity was significantly higher among individuals with secondary infertility 86(71.1%) compared to those with primary infertility 8 (30.8%) [RR 2.3, 95% CI: 1.3 - 4.2; P = 0.005]. Table 4 shows that the age at coitarche (COR 3.3), number of sexual partners (COR 4.6), parity (COR 2.2) and previous sexually transmitted infection (COR 3.1) were significantly associated with chlamydia infection in study population (P=0.001). Further analysis using multivariate logistic regression showed that early age at coitarche (AOR 3.5, 95% CI 2.0-6.1, P=0.001), presence of multiple sexual partners (AOR 2.7, 95%CI 1.5-4.9, P=0.001) and previous sexually transmitted infection (AOR 3.2, 95%CI 1.6-6.4, P=0.001) were significantly associated with increased likelihood of chlamydial infection.

**Table 3:** the relationship between type of tubal infertility and chlamydial seropositivity

Variables	Chlamydia Positive (%) n=26	Chlamydia Negative (%) n=121	Relative Risk (95% CI)	p value
**Type of tubal infertility**				
Secondary	86(71.1)	35(28.9)	2.3(1.3 - 4.2)	0.005
Primary	8(30.8)	18(69.2)		

SI- Secondary infertility, PI- Primary infertility

**Table 4:** multivariate logistic regression analysis of risk factors associated with chlamydia infection among the study population

Variable	Chlamydia positive	Chlamydia negative	COR	P value	AOR	P value	CI
**Age at Coitarche**							
10-19	91(69.5)	67(41.1)	3.3	0.001	3.5	0.001	2.0-6.2
20-29	40(30.5)		Ref				
**Number of lifetime sexual partner**							
Multiple	108(82.4)	82(50.3)	4.6	0.001	2.7	0.001	1.5-4-9
Single	23(17.6)	81(49.7)	Ref				
**Parity**							
Nullipara	68(51.9)	54(33.1)	2.2	0.001	1.4	0.265	0.8-2.3
Primipara/Multipara	63(48.1)	109(66.9)	Ref				
**Previous STD**							
Yes	41(31.3)	21(12.9)	3.1	0.001	3.2	0.001	1.6-6.4
No	90(68.7)	142(87.1)	Ref				

COR- Crude Odds Ratio; AOR- Adjusted Odds Ratio

## Discussion

*Chlamydia trachomatis* infection is an important aetiological factor for pelvic inflammatory disease worldwide, and is often associated with tubal factor infertility [21,22]. The tubal damage associated with chlamydia infection is mostly due to persistence of such infections rather than single acute episodes [19]. This study shows that women with chlamydia IgG seropositivity were two times more likely to have tubal infertility that those who were seronegative. These findings suggest that chlamydia antibody testing could be used as an indirect tool for assessing tubal patency when evaluating infertile patients. Majority of the patients were within the age group of 21 to 30 years, similar findings of 66.0% and 74.8% were reported in Abakalki and Uganda [23,24]. This could be attributed to the fact that this age group coincides with the active reproductive age of general population. Majority (60%) of women with tubal infertility were nulliparous, similar to 65% reported in Benin [25]. This might be adduced to the fact that childlessness is associated with psychological and emotional stress in our environment. This makes the nulliparous women to present early and frequently in the gynaecological clinic compared to parous women with infertility.

The prevalence of *Chlamydia trachomatis* antibody among pregnant women was 25.2%. This was comparable with 25.7% in Brazil [26]. However, lower prevalence of 13.2% and 13.3% were reported in Ile -Ife and Benin, respectively [25,27]. These differences in the prevalence rates may be due to sociodemographic characteristics, cultural differences and varied social behaviour of the different population studied. The high prevalence among antenatal women calls for special attention to screening and management of *Chlamydia trachomatis* infection in pregnant women to avert maternal and foetal morbidities associated with the infection.

This study shows that 63.9% of the women with tubal infertility were positive for *Chlamydia trachomatis*. This was higher than 18 - 20% reported by World Health Organization [25]. Studies done in other parts of Nigeria showed prevalence rates of *Chlamydia trachomatis* infection ranging from 23.4% to 65.8% among tubal infertile patients [28,29]. Majority of the cases (82%) had secondary infertility, this was in agreement with earlier reports [8,23]. This might be attributed to complications arising from post abortal and postpartum infections. Women with *Chlamydia trachomatis* infection had two times higher risk of having secondary infertility compared to those with primary infertility. Women with secondary infertility had two times higher risk of having *Chlamydia trachomatis* infection compared to those with primary infertility. This trend was similar to the findings of Malik *et al*. [30]. This may imply that *Chlamydia trachomatis* infection plays a more important role in the aetiology of secondary tubal infertility than primary tubal factor infertility. Women who had early coitarche (10 -19 years) had increased likelihood of chlamydia seropositivity compared to women with late coitache (20-29 years). Moreover, some authors have reported that the highest rate of chlamydia infections occur in adolescent females aged between 15 and 19 years [31]. This had led to United States Centre for Disease Control and Prevention recommending screening of all sexually active women aged less than 25 [32].

Study participants with multiple life time sexual partners had two times higher risk of chlamydia infection compared with those with single sexual partner. Moreover, women with previous history of sexually transmitted infections had three times higher likelihood of chlamydia seropositivity than women without such history. This confirms the previously established fact that chlamydia is a very common sexual transmitted infection. The proportion of *Chlamydia trachomatis* seropositivity reported in this study was significantly higher among women with tubal infertility compared to pregnant controls. This was consistent with previous studies done by Omoleye *et al*. [33] and Akande *et al*. [14] in Nigeria. This finding corroborates the widely held scientific view that chlamydia is a frontline organism in the aetiology of tubal blockage [10]. More so, this high prevalence rate of *Chlamydia trachomatis* infection may probably be the reason for high rate of tubal infertility (65%-85%) reported in the country [8].

From the findings of this study, chlamydia immunoglobulin G antibody assay using ELISA technique can be used to triage infertile patients that will require further evaluation with hysterosalpingography and laparoscopy, especially in low resource settings. Apart from the effectiveness, it also averts numerous complications associated with invasive procedures [8]. The limitation of the study is the fact that the diagnosis of chlamydia was not based on polymerase chain reaction which has better accuracy than ELISA. However, this technique is expensive and not readily available in Nigeria. Another limitation is that IgG titration was not done in this study making it difficult to quantify the extent of infection. Abdella *et al*. [34] reported that results of chlamydia immunoglobulin G antibody assay using ELISA technique was not significantly different from that of polymerase chain reaction, and it is relatively cheap. Hence, chlamydia ELISA assay is an excellent option for patients´ evaluation during infertility work up in developing countries like Nigeria where there is a huge burden of tubal factor infertility.

## Conclusion

This study found a significantly higher prevalence of *Chlamydia trachomatis* infection among women with tubal factor infertility compared to pregnant control. Furthermore, early age of coitarche, multiple sexual partners and previous sexually transmitted infections were significantly associated with increased risk of chlamydia seropositivity. It is thus very expedient to create awareness and enlighten young women on risk factors associated with *Chlamydia trachomatis* infection. Sexually active individuals should have routine screening for *Chlamydia trachomatis* infection done and those found to be recently infected should be treated to prevent future occurrence of tubal factor infertility. *Chlamydia trachomatis* antibody testing could be used as marker for tubal blockage when evaluating infertile patients.

### What is known about this topic

- *Chlamydia is implicated in tubal infertility;*
- *Secondary infertility is commoner than primary infertility in our environment*.

### What this study adds

- *Significantly higher serum chlamydia antibody among tubal infertile women;*
- *The likelihood of chlamydia infection among individual with risk factors*.
